# Development and evaluation of a deep learning model to reduce exomass-related metal artefacts in cone-beam CT: an *ex vivo* study using porcine mandibles

**DOI:** 10.1093/dmfr/twae062

**Published:** 2024-11-26

**Authors:** Matheus L Oliveira, Susanne Schaub, Dorothea Dagassan-Berndt, Florentin Bieder, Philippe C Cattin, Michael M Bornstein

**Affiliations:** Department of Oral Diagnosis, Division of Oral Radiology, Piracicaba Dental School, University of Campinas, Piracicaba, 13414-903, Brazil; Department of Oral Health & Medicine,University Center for Dental Medicine Basel UZB, University of Basel, Basel, 4058, Switzerland; Department of Biomedical Engineering, Faculty of Medicine, University of Basel, Allschwil, 4123, Switzerland; Department of Oral Health & Medicine,University Center for Dental Medicine Basel UZB, University of Basel, Basel, 4058, Switzerland; Center for Dental Imaging, University Center for Dental Medicine Basel UZB, University of Basel, Basel, 4058, Switzerland; Department of Biomedical Engineering, Faculty of Medicine, University of Basel, Allschwil, 4123, Switzerland; Department of Biomedical Engineering, Faculty of Medicine, University of Basel, Allschwil, 4123, Switzerland; Department of Oral Health & Medicine,University Center for Dental Medicine Basel UZB, University of Basel, Basel, 4058, Switzerland

**Keywords:** cone-beam CT, dental implants, artefacts, artificial intelligence, deep learning

## Abstract

**Objectives:**

To develop and evaluate a deep learning (DL) model to reduce metal artefacts originating from the exomass in cone-beam CT (CBCT) of the jaws.

**Methods:**

Five porcine mandibles, each featuring six tubes filled with a radiopaque solution, were scanned using four CBCT units before and after the incremental insertion of up to three titanium, titanium-zirconium, and zirconia dental implants in the exomass of a small field of view. A conditional denoising diffusion probabilistic model, using DL techniques, was employed to correct axial images from exomass-related metal artefacts across the CBCT units and implant scenarios. Three examiners independently scored the image quality of all datasets, including those without an implant (ground truth), with implants in the exomass (original), and DL-generated ones. Quantitative analysis compared contrast-to-noise ratio (CNR) to validate artefact reduction using repeated measures analysis of variance in a factorial design followed by Tukey test (*α* = .05).

**Results:**

The visualisation of the hard tissues and overall image quality was reduced in the original and increased in the DL-generated images. The score variation observed in the original images was not observed in the DL-generated images, which generally scored higher than the original images. DL-generated images revealed significantly greater CNR than both the ground truth and their corresponding original images, regardless of the material and quantity of dental implants and the CBCT unit (*P* < .05). Original images revealed significantly lower CNR than the ground truth (*P* < .05).

**Conclusions:**

The developed DL model using porcine mandibles demonstrated promising performance in correcting exomass-related metal artefacts in CBCT, serving as a proof-of-principle for future applications of this approach.

## Introduction

Despite the well-known numerous advantages of cone-beam CT (CBCT), a significant limitation of this imaging modality is its susceptibility to artefacts, which are imperfections in the reconstructed image that do not correspond to the real object, reduce image quality, and can negatively affect certain diagnostic tasks.^1^ Among the various sources of artefact formation in CBCT, objects with high physical density and/or composed of high atomic number chemical elements, such as dental implants, surgical plates, and endodontic filling materials, stand out due to their frequent use in dental treatments and their ability to cause a phenomenon known as X-ray beam hardening.^2^ Following image reconstruction in CBCT, artefacts resulting from X-ray beam hardening directly compromise the assessment of conditions such as bone-implant contact for investigating dental implant osseointegration, the adaptation of metallic restorations, the estimation of the volume of metal objects, and indirectly impact the evaluation of structures and conditions adjacent to metals including per-implant bone loss.^1^^,^^3^^,^^4^

The use of small fields of view (FOV) in CBCT whenever possible is currently recommended as it indirectly contributes to increased image sharpness associated with a relative reduction in the radiation dose to which the patient is exposed.^5^ However, FOVs with a reduced diameter indirectly increase the exomass—the area located outside the FOV that interacts with the radiation beam as it is situated between the radiation source and the image receptor. Quantitative studies published recently have shown that the presence of metallic materials in the exomass produces irreparable artefacts in CBCT images. These artefacts, resulting from multiple contributing effects, reduce the average grey values and increase image noise, are exacerbated when combined with motion artefacts, and are more prominent in the central region of the FOV.^6^^,^^7^ Such artefacts have been shown to be resistant to reduction through metal artefact reduction algorithms,^8^ which are tools that demonstrated satisfactory efficacy for metal objects located within the FOV, also referred to as endomass, in laboratory setups^2^ but exhibit limited efficacy in preclinical studies.^9^ In dentistry, patients often require CBCT examinations to assess a structure or region adjacent to dental implants and/or crowns, restorations, or metal plates that may eventually be in the exomass. Therefore, strategies for reducing metal artefacts originating from the exomass are a clinical necessity.

Artificial intelligence has made remarkable advancements and has found multiple applications in dental imaging, including those related to diagnosis, treatment planning, forensic imaging, and image processing and reconstruction.^10–12^ Deep learning (DL), a subset of artificial intelligence, has already shown promising results for various tasks, such as mandibular canal segmentation,^13^ tooth segmentation,^14^ detection of impacted third molars,^15^ aiding in CBCT interpretation by students,^16^ and, most importantly, artefact correction in CBCT scans.^17^^,^^18^ Among the types of DL models, denoising diffusion probabilistic models (DDPMs) are state-of-the-art generative models that employ a sequential denoising process to map a normal distribution to a data distribution, allowing for the sampling of high-fidelity outputs akin to the original noise-free samples.^19^^,^^20^

Considering the inevitable interaction of the X-ray beam with highly attenuating materials when present in the exomass, the subsequent deterioration of CBCT image quality, and the continuous development of new DL models and applications in recent years, the objective of this study was to develop and evaluate a DL model to reduce metal artefacts originating from the exomass in CBCT in a preclinical model.

## Methods

### Imaging phantom preparation

Five fresh porcine mandibles were obtained with preserved soft tissues. Each mandible underwent the extraction of three teeth: the first premolar and second molar on one side and the second premolar on the opposite side. The selection of these teeth was based on their anatomical position to ensure that the space between them could accommodate a FOV with a 5 cm diameter. The sides chosen for extraction were alternated such that three porcine mandibles followed one configuration, and two porcine mandibles followed a mirrored configuration.

In the extraction sites, 15 mm deep implant beds were prepared, beginning with a Ø 2.3 mm round bur (Straumann, Basel, Switzerland), followed by a progressive sequence from Ø 2.2 mm pilot drills (Straumann) to Ø 4.8 mm twist drills (Straumann). Those perforations were intended to be slightly larger than the diameter of the 4.1 mm dental implants to be inserted so that they could be easily placed and removed without the need of moving the porcine mandible between CBCT scans.

**Figure 1.**
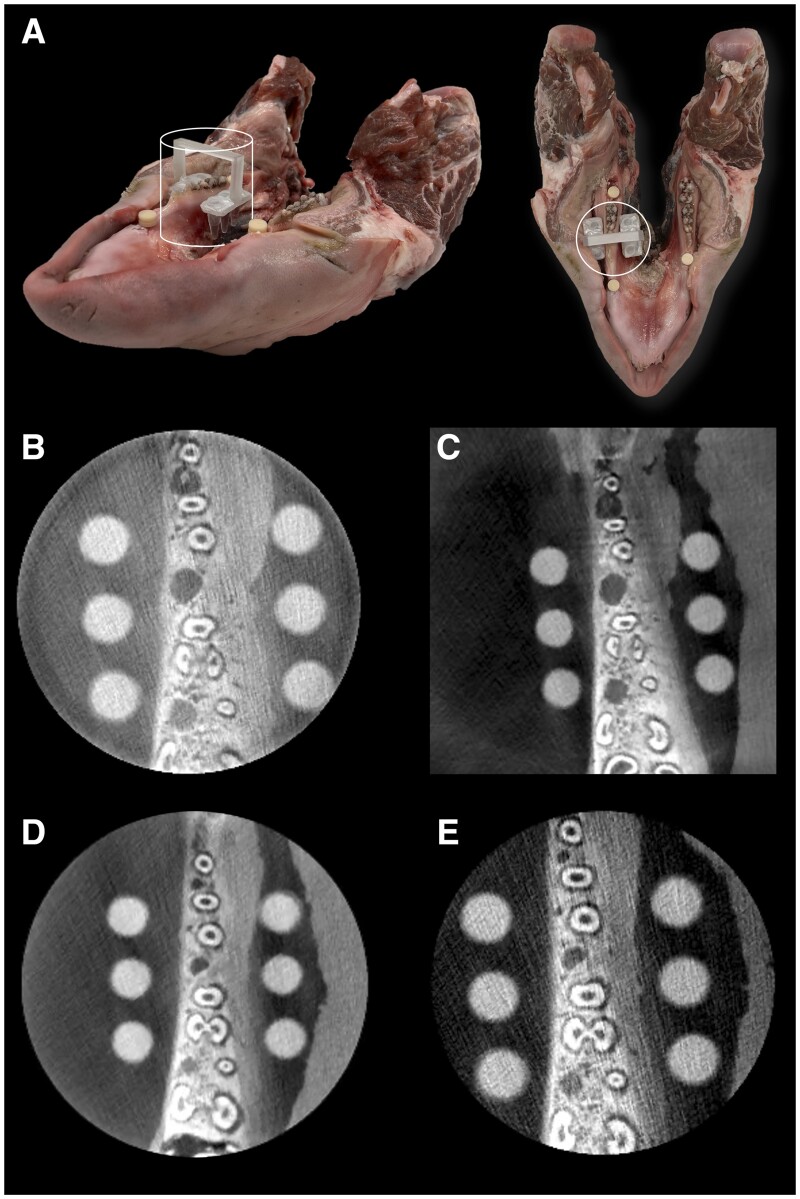
Ground truth setup (no dental implant in the exomass). (A) Lateral and top photographs of the porcine mandible: the superimposed empty shapes represent the small field of view. The resinous plugs, used to prevent clogging of the implant perforation, were removed during the CBCT scanning. Representative CBCT axial reconstructions from four different units: (B) 3D Accuitomo 170, (C) Axeos, (D) ProMax 3D Max, (E) X800.

An 800 mg mL^−1^ K_2_HPO_4_ aqueous solution was prepared to completely fill six 0.2 mL polypropylene tubes. This is a highly homogeneous radiopaque solution that has been used for quantitative analysis of the grey levels in previous studies.^6^ The tubes were secured into a resinous holder, which was designed using software for computer-aided design (Shapr3D, Budapest, Hungary) and manufactured using the P20 + 3D printer (Straumann) to ensure stability in the posterior region of the porcine mandibles. Three tubes were positioned adjacent to the buccal aspect of the teeth, and three to the lingual aspect (Figure 1A).

### CBCT scanning procedures

Each porcine mandible, accompanied by the six polypropylene tubes, was individually scanned using small and large FOVs from four CBCT units: 3D Accuitomo 170 (Morita, Kyoto, Japan), Axeos (Dentsply-Sirona, Charlotte, NC, United States), ProMax 3D Max (Planmeca, Helsinki, Finland), and X800 (Morita). The tube current and voltage were, respectively, 7 mA and 85 kVp, for all CBCT units. The FOV and voxel sizes are listed in Table 1. The small FOV was centred in the molar region, encompassing the six tubes and ensuring that the three dental implant perforations were situated within the exomass, whereas the large FOV completely encompassed the three dental implants, six tubes, and most of the porcine mandible. Both the small and large FOVs were used for further training of the DL model to increase the quantity and heterogeneity of data, aiming for better generalisation of the model.

**Table 1.** Field of views and voxel sizes (in mm) selected as a function of the CBCT unit.

CBCT unit	Field of view	Voxel
3D Accuitomo 170	40 × 40	0.08
	100 × 50	0.16
Axeos	50 × 50	0.08
	100 × 50	0.25
ProMax 3D Max	50 × 55	0.075
	100 × 55	0.15
X800	40 × 40	0.08
	150 × 50	0.32

An initial CBCT scan was obtained without any dental implant, serving as the ground truth for the absence of exomass-related metal artefacts (Figure 1B-E). This was followed by nine subsequent scans, each involving the insertion of one, two, and three dental implants (4.1 mm in diameter 14 mm in length) made of the same material: either titanium (Ti, Loxim, Straumann), 85% titanium-15% zirconium alloy (Roxolid, Loxim, Straumann), or zirconia (ZrO_2_, Straumann).

With the aid of a scout image, the dental implants and six polypropylene tubes were vertically positioned to be at the same spatial height, thus interacting with the X-rays along the same *z*-axis. Importantly, the dental implants were alternated without moving the porcine mandible or the tubes, ensuring highly controlled exposure geometry. Furthermore, when one or two dental implants were present, their location in the three perforations was randomly determined to achieve a heterogeneous sample. This resulted in a total of 400 CBCT datasets [five porcine mandibles × four CBCT units × two FOVs × (three dental implant materials × three dental implant quantities + no dental implant)], which were exported and stored as Digital Imaging and Communications in Medicine (DICOM) files.

### DL model development

All DICOM files were converted to Neuroimaging Informatics Technology Initiative (NIfTI) format to facilitate the workflow and were pre-processed to prepare them for the DL model.

First, the axial slices of all reconstructed volumes were resized to 256 × 256 pixels. This size was chosen to fit the DL model optimisation into the memory of the available graphics processing unit (GPU). Due to significant variations in background grey values among images from the four CBCT units, adjustments were made accordingly. For 3D Accuitomo 170 and X800, background intensity values below −1000 were clipped, and the top one percentile of intensities was also clipped. For Axeos and Planmeca, both the top and bottom one percentile of the grey values were removed. These adjustments aimed to normalise the grey values across all images to a comparable range. Subsequently, the grey values were normalised to the range [0, 1] as this is the convention used for the DDPM. The slices at the bottommost and topmost positions, where no artefacts were visible, were excluded from the training dataset.

A DDPM was trained to generate artefact-reduced images.^19^^,^^20^ It is a state-of-the-art 2D DL model for image generation and processing, based on an iterative noising and denoising process. Specifically, a conditional DDPM was trained using paired images without a dental implant (ground truth) and with dental implants to perform image-to-image translation. The implementation followed the approach outlined in a previous study.^21^ The DDPM architecture followed a U-Net with attention and timestep-embedding, and hyperparameters based on another previously published study.^22^

The training set included 70 920 axial images originating from four porcine mandibles and with small and large FOV sizes. The test set comprised 9580 axial images originating from the fifth porcine mandible and only the small FOV size since the present study’s focus was on exomass-related metal artefacts. Both datasets included images from all CBCT units and dental implant conditions. Because DDPMs generate data by reversing a noising process, they are highly resistant to overfitting, even when trained on a relatively small dataset,^19^^,^^20^ as was the case in this study.

For data augmentation, random horizontal and vertical flips were applied to the training images. By comparing the intensity profiles of the generated images with their corresponding ground truth counterparts in the validation set, it was determined that the model effectively learned both pixel values and contrast variations present in the ground truth images; therefore, intensity-based data augmentation techniques were not incorporated in this study. The model was trained for 50 epochs with a batch size of 16 on an NVIDIA Quadro RTX 6000 GPU, which took approximately one and a half days. During training, the validation loss was monitored, and the number of epochs was chosen to ensure model convergence. After training the model, slices of all images in the test set were generated, denormalised, and stacked into 3D images in the NIfTI format. Since the slices were generated individually, minute variations in grey values across slices were visible.

### Qualitative evaluation of the DL model

**Figure 2.**
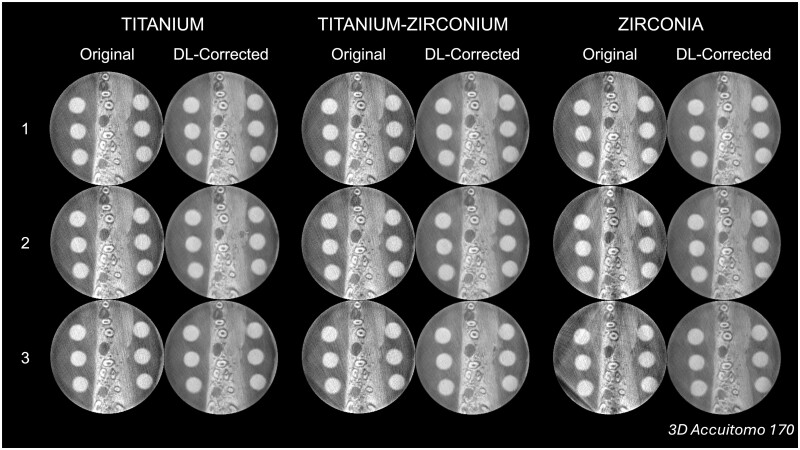
Representative original and deep learning (DL)-corrected CBCT reconstructions at the same axial level as a function of the material and quantity of dental implants in the exomass from the 3D Accuitomo 170 CBCT unit.

**Figure 3.**
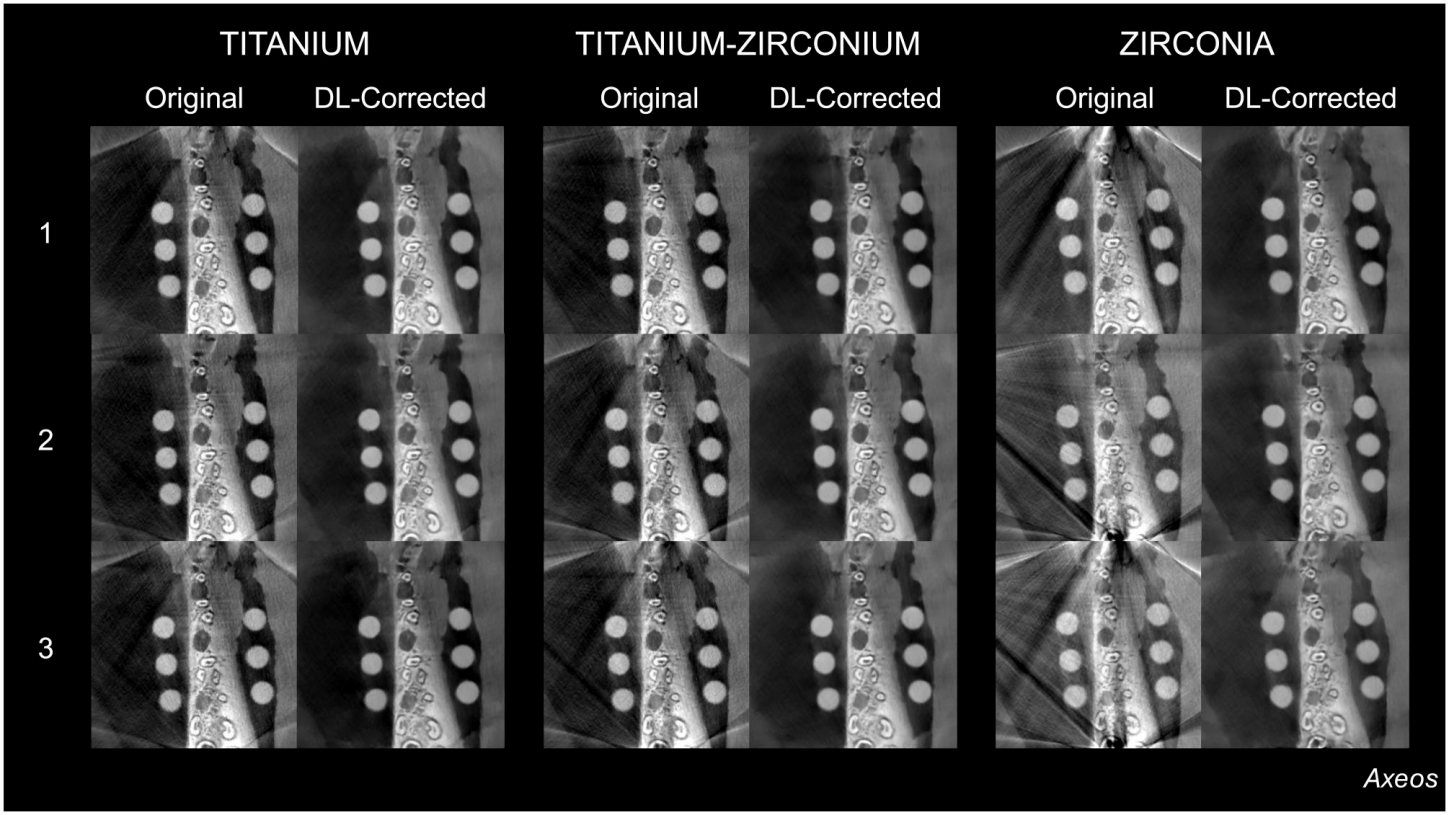
Representative original and deep learning (DL)-corrected CBCT reconstructions at the same axial level as a function of the material and quantity of dental implants in the exomass from the Axeos CBCT unit.

**Figure 4.**
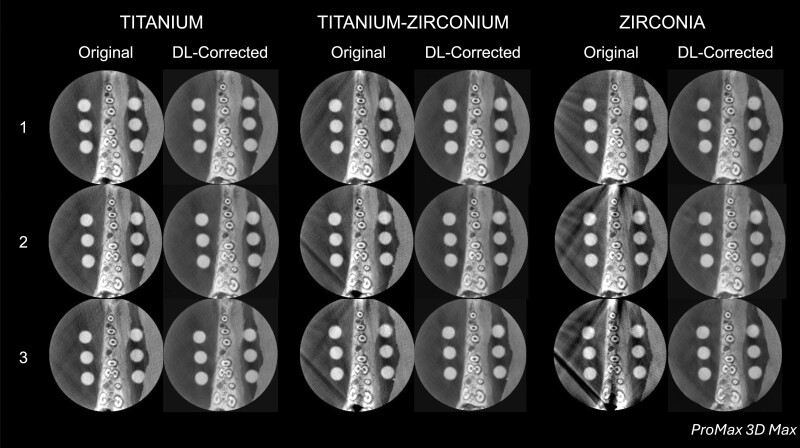
Representative original and deep learning (DL)-corrected CBCT reconstructions at the same axial level as a function of the material and quantity of dental implants in the exomass from the ProMax 3D Max CBCT unit.

**Figure 5.**
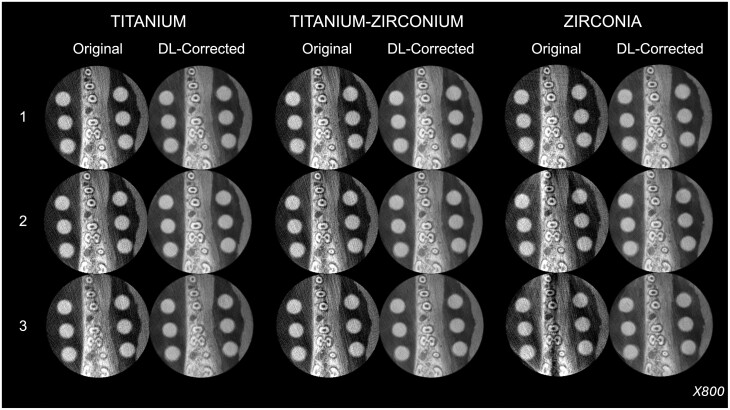
Representative original and deep learning (DL)-corrected CBCT reconstructions at the same axial level as a function of the material and quantity of dental implants in the exomass from the X800 CBCT unit.

Axial images at three levels from each stacked NIfTI file in the test set were exported in the Tagged Image File Format (TIFF). The ground truth, images with implants in the exomass (henceforth referred to as original images), and DL-corrected images at the same axial level and from the same CBCT units were cross-merged into three pairs in a randomised order and without image compression. This resulted in a total of 324 pairs (three pairs × three axial levels × four CBCT units × three dental implant materials × three dental implant quantities). Representative axial images from the datasets containing dental implants are present in Figures 2-5.

Three dentomaxillofacial radiologists independently evaluated all pairs of images and assigned a relative score to each based on their confidence level regarding the deterioration caused by artefacts on the visualisation of (1) the hard tissues and (2) overall image quality. This score derived from the following five-point scale: (1) the image quality on the left is definitely superior to the one on the right, (2) the image quality on the left is possibly superior to the one on the right, (3) the image quality on the left equals the one on the right, (4) the image quality on the right is possibly superior to the one on the left, and (5) the image quality on the right is definitely superior to the one on the left.

The examiners were granted permission to manipulate parameters such as brightness, contrast, and zoom levels, and were blinded to the presence and/or material of the dental implants and the four CBCT units under consideration. To mitigate potential visual fatigue, a maximum of 50 pairs were assessed per session. Following a 15-day interval after the completion of the initial assessment, a randomly selected 25% subset of pairs from each experimental group underwent re-evaluation to assess reproducibility.

### Quantitative evaluation of the DL model

Mean and SD of the grey values were collected from seven circular regions of interest (ROIs; 25 mm in diameter) from 40 axial images where part of the dental implants was present in the exomass of the ground truth, original images with implants in the exomass, and DL-corrected datasets. Six ROIs were selected within the radiopaque homogeneous solution, each within a different polypropylene tube, and one ROI was in a region corresponding to air.

Contrast-to-noise ratio (CNR) was individually calculated for each of the six tubes per axial image and subsequently averaged. The CNR calculation was based on the difference of mean grey values between the tube of interest and the air, divided by the average of the SD values from the same tube of interest and the air, as follows:


CNR = Mean grey value (tube) − Mean grey value (air)Standard deviation (tube) + Standard deviation (air)2

### Data analysis

For the qualitative evaluation of the DL model, the scores assigned by the three examiners were split into two groups: those when the comparisons were between the ground truth and the experimental groups and those when the comparisons were between the original and DL-corrected images within the experimental groups.

Mean and SD values of the scores were calculated including the three examiners, three axial levels, and four CBCT units as a function of the material and quantity of dental implants in the exomass. The obtained values from the comparison of the ground truth with the experimental groups were plotted in radar charts and those comparing original images with their corresponding DL-corrected images were plotted in a 100% stacked bar graph for each material and quantity of dental implants in the exomass. The Kappa test was used to evaluate intra- and inter-examiner reliability, and the resulting coefficients were interpreted according to the classification proposed by Landis & Koch.^23^

For the quantitative evaluation of the DL model, CNR values were compared among the ground truth, original images with dental implants in the exomass, and DL-corrected images, using repeated measures analysis of variance (ANOVA) in a factorial design [two image types (original and DL-corrected) × three materials (Ti, Ti-Zr, and ZrO_2_) × three quantities (1, 2, and 3) + ground truth]. Pairwise comparisons were conducted using *post hoc* Tukey test. Statistical analyses were conducted using Jamovi software (version 2.3.28; JASP project; available at www.jamovi.org), with a significance level of 5% (*α* = .05).

## Results

**Figure 6.**
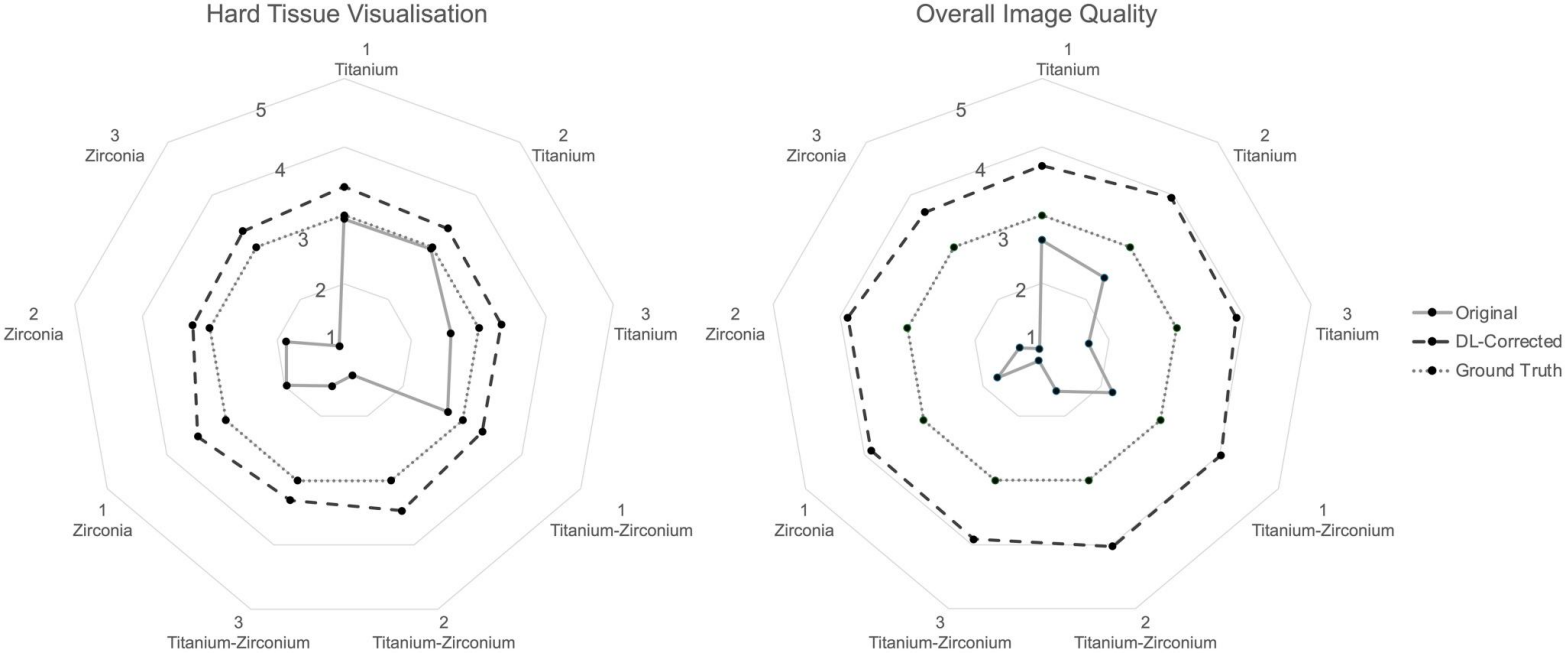
Radar charts of the average scores for hard tissue visualisation and overall image quality for both original and deep learning (DL)-corrected images in the presence of dental implants made from three materials and in three quantities in the exomass for the four CBCT units, compared to the ground truth (no dental implant in the exomass). Scores higher than the ground truth (outer from score 3) indicate improved conditions, while scores lower than the ground truth (inner from score 3) indicate worsened conditions.

In most cases, the perceived visualisation of the hard tissues was reduced in the original images and increased in the DL-corrected images compared to the ground truth. This trend was similar and even more noticeable for the perceived overall image quality. The considerable variations observed in the perceived visualisation of the hard tissues and overall image quality among the different materials and quantities of dental implants in the exomass of the original images were not observed in the DL-corrected images, whose scores were mostly distributed around 4 (possibly superior to the ground truth). Radar charts in Figure 6 graphically illustrate this variation. Outer values from score 3 represent improved qualitative evaluation, while inner values indicate a worsened qualitative evaluation.

When compared to the original images, DL-corrected images generally received higher scores. However, the discrepancy between them was more pronounced for zirconia and at a higher number of dental implants in the exomass. As shown in Table 2, the examiners’ confidence level in this perception was greater for overall image quality than for hard tissue visualisation, with a more visible shift of the scores to the right (score 5; DL-corrected image quality was definitely superior to the original images). Intra-examiner agreement demonstrated almost perfect strength and inter-examiner agreement showed substantial strength, indicated by Kappa coefficients of 0.94 and 0.79, respectively.

**Table 2.** Mean ± SD of the scores assigned by the radiologists for hard tissue visualisation and overall image quality between original and deep learning (DL)-corrected CBCT images as a function of the material and quantity of dental implants in the exomass.

Material	Quantity	Hard tissue visualisation	Overall image quality
Titanium	1	3.6 ± 0.7	4.1 ± 0.7
	2	3.7 ± 0.6	4.6 ± 0.6
	3	3.8 ± 0.7	4.4 ± 0.5
Titanium-Zirconium	1	3.6 ± 0.8	4.5 ± 0.5
	2	4.0 ± 0.6	4.8 ± 0.4
	3	4.6 ± 0.6	4.9 ± 0.2
Zirconia	1	4.2 ± 0.7	4.7 ± 0.5
	2	4.8 ± 0.4	4.9 ± 0.4
	3	5.0 ± 0.2	5.0 ± 0.0

Scores higher than the 3 (when original equals DL-corrected images) indicate a preference for DL-corrected images.

As shown in Table 3, DL-corrected images revealed significantly greater CNR values than both the ground truth and their corresponding original images, regardless of the material and quantity of dental implants and the CBCT unit (*P* < .05). Furthermore, in general, original images revealed significantly lower CNR values than the ground truth (*P* < .05).

**Table 3.** Mean ± SD of contrast-to-noise ratio (CNR) from ground truth (no dental implant in the exomass) and original and deep learning (DL)-corrected images in the presence of dental implants made of three materials and at three quantities in the exomass for the four CBCT units.

CBCT unit		Titanium		Titanium-zirconium		Zirconia	
		Original^b^	DL-corrected	Original^b^	DL-corrected	Original^b^	DL-corrected
3D Accuitomo 170	1	12.8 ± 0.8	19.7^a^ ± 1.2	11.8^a^ ± 0.8	17.0^a^ ± 1.3	12.0^a^ ± 0.7	19.5^a^ ± 1.3
	2	12.1^a^ ± 0.7	18.2^a^ ± 1.6	12.2^a^ ± 0.7	16.4^a^ ± 1.3	12.0^a^ ± 0.8	18.4^a^ ± 1.6
	3	10.9^a^ ± 0.6	16.1^a^ ± 1.8	7.8^a^ ± 0.6	16.9^a^ ± 1.1	10.7^a^ ± 0.6	18.0^a^ ± 1.2
	Ground truth	13.3 ± 0.9					
Axeos	1	25.7 ± 2.8	52.2^a^ ± 5.8	26.2^a^ ± 1.7	54.4^a^ ± 6.7	17.4^a^ ± 2.3	37.2^a^ ± 8.7
	2	23.4^a^ ± 2.1	52.7^a^ ± 6.7	22.9^a^ ± 2.7	39.8^a^ ± 5.3	17.1^a^ ± 1.4	39.8^a^ ± 7.6
	3	23.1^a^ ± 2.6	49.4^a^ ± 6.7	13.3^a^ ± 1.6	45.4^a^ ± 7.0	12.1^a^ ± 1.7	26.2^a^ ± 4.4
	Ground truth	28.2 ± 1.8					
ProMax 3D Max	1	15.5^a^ ± 1.8	24.2^a^ ± 3.2	16.4^a^ ± 0.6	24.2^a^ ± 1.6	9.2^a^ ± 0.6	24.3^a^ ± 2.8
	2	14.7^a^ ± 1.0	13.3^a^ ± 0.9	13.7^a^ ± 0.9	19.7^a^ ± 1.8	9.4^a^ ± 0.3	20.1^a^ ± 2.5
	3	13.4^a^ ± 1.2	21.7^a^ ± 2.3	8.3^a^ ± 0.7	21.9^a^ ± 2.4	8.4^a^ ± 0.3	22.1^a^ ± 2.1
	Ground truth	16.9 ± 1.0					
X800	1	9.8 ± 0.2	19.4^a^ ± 0.7	8.9^a^ ± 0.2	17.0^a^ ± 0.6	8.7^a^ ± 0.2	16.0^a^ ± 0.5
	2	8.0^a^ ± 0.1	15.9^a^ ± 0.6	8.6^a^ ± 0.2	14.4^a^ ± 0.6	7.5^a^ ± 0.1	14.7^a^ ± 0.6
	3	8.2^a^ ± 0.1	15.9^a^ ± 0.5	5.8^a^ ± 0.1	15.5^a^ ± 0.5	7.0^a^ ± 0.1	14.6^a^ ± 0.5
	Ground truth	9.4 ± 0.1					

a Significantly different from ground truth (*P* < .05).

b Significantly different from DL-corrected for all values in the column (*P* < .05).

## Discussion

Several attempts have been made to overcome the image deterioration imposed by metal artefacts in CBCT. These attempts include varying X-ray exposure parameters,^24^ applying image post-processing algorithms,^2^ and, more recently, exploring artificial intelligence models.^18^^,^^25^^,^^26^ Typically, laboratory or clinical experiments have focused on scenarios where the artefact-generating metal is in the FOV. However, a recent study revealed unsatisfactory performance of metal artefact reduction algorithms when the artefact-generating metal is in the exomass, which is the area outside the FOV but still between the X-ray tube and the image sensor.^8^ This underscores the importance of finding solutions for this specific situation, given the high frequency of use of small FOVs and the widespread application of materials with high potential for X-ray beam hardening, such as dental implants and metallic restorations, in oral rehabilitation. In line with this, this study aimed at developing a unique DL model, which revealed a promising capacity for correcting exomass-related metal artefacts, as identified by both qualitative and quantitative analysis.

A DDPM was the core technique of the present DL approach. It is a cutting-edge DL model consisting of a customised iterative sequence involving introduction and removal of noise. The likelihood of such interference depends on the previously achieved state to generate samples that match the data within finite time.^19^^,^^20^^,^^22^ To accommodate this approach and generate paired images, a unique methodological design was employed, wherein CBCT scans were acquired with the object of interest consistently positioned. Each porcine mandible was placed and stabilised within the CBCT unit, enabling the series of scans of the ground truth and all dental implant scenarios without repositioning. This was made feasible by preparing implant beds with dimensions slightly larger than the 4.1 mm implant diameter, facilitating delicate placement and removal of the implants. Furthermore, to simulate a wide range of clinical scenarios, experimental groups were composed of combinations of three materials and three quantities of dental implants, in addition to using four different CBCT units. As depicted in Figures 2-5, this resulted in varying degrees of artefact expression, which is beneficial for DL training as it enhances the generalisability of the resulting model.

The perceived performance of the developed DL model in relation to the original images containing dental implants in the exomass was improved in all simulated scenarios. However, when comparing original and DL-corrected images to the ground truth, it is noticeable that DL-corrected image quality reached a consistent level across the simulated scenarios, whereas the original images varied prominently. Such a variation among original images may strongly relate to the direct relationship between X-ray beam hardening and the atomic number of the main chemical elements composing the implants (Ti, Z = 22; Zr, Z = 40). Although a previous study did not detect significant differences in CBCT-based identification of peri-implant defects using the same implant materials as those tested in this study,^27^ the authors reported that zirconia had a greater impact regarding the generated artefacts on defect diagnosis than titanium and titanium-zirconium alloy implants. Furthermore, zirconia implants also resulted in a larger underestimation of the width of peri-implant defects than for implants with titanium and titanium-zirconium alloy implants. Another investigation assessing different materials in the exomass observed greater artefact impact from amalgam compared to cobalt-chromium alloy and titanium.^6^ Importantly, the artefact expression of titanium-zirconium alloy and zirconia implants in the exomass has not yet been investigated in detail.

Regarding the quantitative analysis, CNR also demonstrated the value of the developed DL model. CNR is a frequently used approach in quantitative image analysis, as it scales two key image quality parameters: contrast and noise.^28^ When comparing two homogeneously distinct areas, higher contrast with a low average SD of their grey values is related to better image quality, as this suggests that contrast outweighs image noise. Interestingly, both qualitative and quantitative metrics revealed that besides reducing exomass-related metal artefacts, the proposed DL model also produced images superior to the ground truth, possibly due to the inherent noise reduction capability of the DDPM. Importantly, the inclusion of tubes containing radiopaque solutions in the FOV was essential for quantitative analysis as they served as highly controlled references for grey value evaluation. Rather than a limitation, the tubes facilitated the initial validation of this proof-of-principle study. Future work should expand to more diverse datasets, including scans without tubes and those obtained from patients under clinical conditions, to improve generalizability.

Despite the rather satisfactory performance of the proposed DDPM model, it is important to highlight that it was developed based on individually exported axial slices rather than the 3D dataset as a volume. Therefore, greyscale variation was visible across cross-sectional slices, underscoring the need for further investigations into and development of a volumetric approach. Moreover, a more advanced method to overcome exomass-related metal artefacts could involve exploring the raw projection images prior to primary reconstructions, as the metals in the exomass will certainly be visible in some projection images. The challenge in this context would be obtaining all the geometry parameters used for image reconstruction, which are not typically disclosed by manufacturers. Operating in the image domain offers the advantage of scanner-independence, facilitating integration into processing pipelines and enhancing the model's suitability for clinical applications.

The primary goal of this study was to establish and validate the model's core principles and effectiveness in addressing specific artefacts. While higher-resolution inputs are ideal for clinical applications, utilizing lower-resolution data allows for practical experimentation within the constraints of available GPU memory and computational resources. The trade-off between resolution and computational feasibility is a common challenge in DL. Furthermore, DDPMs have demonstrated the ability to generate high-quality images at a resolution of 512 × 512 pixels, with the model architecture operating in pixel space,^29^ as employed in the present study. Consequently, it is plausible to upscale the model to produce higher-resolution images; however, limited resources remain a constraint.

In conducting an *ex-vivo* study using porcine mandibles, the present research encountered limitations in accurately simulating potential real-life interferences, such as involuntary movements during CBCT scans in living patients. Nonetheless, due to the inherent biological risks associated with X-ray exposure, the experimental model employed in this study facilitated the completion of 80 CBCT scans of the same mandible in a highly stable position. This stability was essential for the pairwise development of the DL model and was verified by comparing image pairs of the same mandible through difference maps, which confirmed that the images were sufficiently registered. The use of porcine mandibles for this study was chosen over human mandibles, as they allow for the attainment of the current research objectives while avoiding unnecessary perforation of human cadaver tissues, which could raise ethical concerns and complicate Institutional Review Board approval. Moreover, despite anatomical variations between porcine and human mandibles, all assessments conducted herein were internally comparative, enhancing the potential applicability of findings to diverse contexts.

Although the present DL model was trained on CBCT images from a limited sample of four porcine mandibles, anatomical variation across slices, data augmentation techniques, and the use of different CBCT units, dental implant materials and quantities enhance the robustness of the study, reduce redundancy, and help prevent overfitting. Importantly, the validation slices, sourced from a mandible excluded from the training set, showed strong performance, demonstrating the model's ability to generalize effectively. Additionally, DDPMs generate training data progressively by learning to reverse a noising process, a structure that makes them highly resistant to overfitting, even with relatively small datasets.^19^^,^^20^ Because artificial intelligence is a rapidly evolving field, foundational research at this stage is essential in shaping its future development and potential clinical application. Proof-of-principle studies are not only valuable but necessary, as they demonstrate feasibility, uncover challenges, and guide future advancements.

In conclusion, the developed DL model demonstrated promising performance in correcting exomass-related metal artefacts in CBCT. Further studies exploring its feasibility for clinical CBCT scans and further developments of volumetric approaches are encouraged.
